# Identification of a TGF-β signaling-related gene signature for prediction of immunotherapy and targeted therapy for lung adenocarcinoma

**DOI:** 10.1186/s12957-022-02595-1

**Published:** 2022-06-06

**Authors:** Qian Yu, Liang Zhao, Xue-xin Yan, Ye Li, Xin-yu Chen, Xiao-hua Hu, Qing Bu, Xiao-ping Lv

**Affiliations:** 1 Department of Oncology, the First Affiliated Hospital of Guangxi Medical University, Guangxi Zhuang Autonomous Region, No. 6 Shuangyong Rd, Nanning, 450100 China; 2 Department of Gastroenterology, the First Affiliated Hospital of Guangxi Medical University, Guangxi Zhuang Autonomous Region, No. 6 Shuangyong Rd, Nanning, 450100 China

**Keywords:** TGF-β signaling, Lung adenocarcinoma, Risk model, Prognosis, Tumor microenvironment, Immunotherapy

## Abstract

**Background:**

Transforming growth factor (TGF)-β signaling functions importantly in regulating tumor microenvironment (TME). This study developed a prognostic gene signature based on TGF-β signaling-related genes for predicting clinical outcome of patients with lung adenocarcinoma (LUAD).

**Methods:**

TGF-β signaling-related genes came from The Molecular Signature Database (MSigDB). LUAD prognosis-related genes were screened from all the genes involved in TGF-β signaling using least absolute shrinkage and selection operator (LASSO) Cox regression analysis and then used to establish a risk score model for LUAD. ESTIMATE and CIBERSORT analyzed infiltration of immune cells in TME. Immunotherapy response was analyzed by the TIDE algorithm.

**Results:**

A LUAD prognostic 5-gene signature was developed based on 54 TGF-β signaling-related genes. Prognosis of high-risk patients was significantly worse than low-risk patients. Both internal validation and external dataset validation confirmed a high precision of the risk model in predicting the clinical outcomes of LUAD patients. Multivariate Cox analysis demonstrated the model independence in OS prediction of LUAD. The risk model was significantly related to the infiltration of 9 kinds of immune cells, matrix, and immune components in TME. Low-risk patients tended to respond more actively to anti-PD-1 treatment, while high-risk patients were more sensitive to chemotherapy and targeted therapy.

**Conclusions:**

The 5-gene signature based on TGF-β signaling-related genes showed potential for LUAD management.

**Supplementary Information:**

The online version contains supplementary material available at 10.1186/s12957-022-02595-1.

## Background

Lung cancer is the second most commonly diagnosed cancer and a leading cause of cancer death in 2020, accounting for approximately 11.4 and 18.0% of all cancer cases and cancer of the year [1]. Lung adenocarcinoma refers to a type of lung cancer resulted from epithelial cells of glands or adenoid structures and is the most frequently diagnosed subtype in patients who do not have a smoking habit [2]. Though LUAD treatment has been greatly improved, its 5-year survival rate is approximately 15% [3]. Some studies have shown that biomarker-driven treatment can improve the survival rate of patients with advanced and metastatic LUAD [5–7]. Earlier, Zhang et al. [8] found that the high expression of CCT6A is related to the poor prognosis of non-small cell lung cancer (LUSC). Li et al. [9] reported that moesin can be used as a prognostic marker of lung adenocarcinoma, and it improves patients’ prognosis by enhancing immune lymphocyte infiltration. Cai et al. [10] identified TCN1 as a prognostic and diagnostic biomarker for lung adenocarcinoma by using a variety of bioinformatics methods. Similarly, Wu et al. [11] identified DPYSL2 as a diagnostic and prognostic potential in LUAD and an immunotherapeutic target based on a variety of public databases and verified that it is effective to screen biomarkers of single gene of lung adenocarcinoma by using public database data. In addition, a multi-gene joint model has also achieved great success. Jia et al. [12] used m6A-related gene set to determine 3-gene signature using various bioinformatics methods to evaluate the prognosis and immune response of LUSC. Xu et al. [13] applied protein interaction network mining to identify seven key genes in LUAD and validated that the high expression of these genes is related to adverse prognosis and may improve the response to immunotherapy. Peng et al. [14] integrated gene expression and mutation characteristics and developed a 14-gene prognostic model to evaluate tumor progression in LUAD. Although there have been a large number of biomarkers, they are rarely used in clinic practice, which suggests that effective biomarkers need to be further verified to better guide the treatment of LUAD.

The transforming growth factor (TGF)-β signaling pathway plays a dual role in tumorigenesis. In early cancer cells, the TGF-β signaling pathway could inhibit tumor growth and promote cell cycle arrest and apoptosis. However, its activation in advanced cancer stimulates tumorigenesis, facilitating cancer cell escape from immune surveillance and inducing metastasis and chemical resistance [15]. TGF-β signaling inhibition is an emerging strategy in cancer therapy, several small and large molecule compounds have been developed to inhibit TGF-β signaling [16]. For example, TGF-β antibodies, antisense oligonucleotides, and small molecules inhibitors of TGF-β receptor-1 (TGF-βR1) have shown great potential in inhibiting TGF-β signaling [17, 18]. Recent development of the TGF-β signaling pathway with related gene expression prognostic tools and response biomarkers may provide alternative means to select patients suitable for receiving the anti-TGF-β intervention [19]. However, at present, the development of effective TGF-β signaling inhibitors faces many clinical challenges, especially deciding the timing of treatment and selecting effective biomarkers for patient selection [20].

In the precision oncology era, new predictive tools have been developed to study tumor progression at a molecular level, providing new possibilities for the development of diagnosis, prognosis, and targeted therapy for cancer management [21]. In this study, 54 TGF-β signaling-related genes were identified, and a prognostic model based on TGF-β signaling-related genes was established and verified in 6 independent meta-cohorts. This study established a prognostic model and applied it to analyze the immune cell infiltration and response of LUAD patients with different risks to immunotherapy, chemotherapy, and targeted therapy. In clinical practice, the prognostic model will help to distinguish LUAD patients who could benefit from receiving TGF-β signaling inhibition treatment.

## Methods

### Acquisition of public data and processing

RNA-seq and corresponding clinical data of LUAD samples, including age, gender, T stage, N stage, M stage, AJCC stage, OS, and smoking history, were downloaded from TCGA (https://tcga-data.nci.nih.gov/tcga/). Transcriptome profiling data of LUAD patients in independent Meta-Cohorts (GSE31210, GSE30219, GSE50081, GSE13213, GSE19188, GSE41271) were downloaded from Gene Expression Omnibus (GEO) database. Patients’ clinical data are listed in Table 1. Supplementary Fig. 1 shows the workflow of this study.

**Table 1:** Clinical characteristics of patients in training and validation sets

Clinical features	TCGA-LUAD	GSE31210	GSE30219	GSE50081	GSE13213	GSE19188	GSE41271
**OS**							
0	318	191	40	76	68	16	112
1	182	35	43	51	49	24	70
**T stage**							
T1	167		69	43			
T2	267		12	82			
T3	45		2	2			
T4	18						
TX	3						
**N stage**							
N0	324		80	94			
N1	94		3	33			
N2	69						
N3	2						
NX	11						
**M stage**							
M0	332		83	127			
M1	24						
MX	144						
**Stage**							
I	268	168		92	79		101
II	119	58		35	13		28
III	80				25		49
IV	25						4
X	8						
**Gender**							
Male	230	121	65		60	25	92
Female	270	105	18		57	15	90
**Age**							
≤ 65	237	176	60	40	78		
> 65	253	50	23	87	39		
NA	10						
**Smoking**							
1	71						
2	119						
3	129						
4	163						
5	4						
7	14						

Annotation: Lifelong non-smoker (less than 100 cigarettes smoked in lifetime) = 1; current smoker (includes daily smokers and non-daily smokers or occasional smokers) = 2; current reformed smoker for > 15 years (greater than 15 years) = 3; current reformed smoker for ≤ 15 years (less than or equal to 15 years) = 4; current reformed smoker, duration not specified = 5; smoking history not documented = 7

### Identification of TGF-β signaling-related genes

The TGF-β signaling-related data set was retrieved from MSigDB (https://www.gsea-msigdb.org/gsea/msigdb/index.jsp) [22], and 54 TGF-β signaling-related genes were identified and collated.

### Prognostic gene signature construction

Univariate Cox analysis was employed to identify genes affecting OS of patients with LUAD from 54 TGF-β signaling-related genes. After that, the prognostic genes were further identified by LASSO and multivariate Cox regression to establish a prediction model. Patients in TCGA and GEO were grouped into low-risk and high-risk groups according to the risk score. The survival status plot, risk heatmap, and Kaplan–Meier curve were employed to compare the survival difference between the two groups. The receiver operating characteristic curve (ROC) was used to evaluate the accuracy and specificity of the model.

### Independent prognostic value analysis

Univariate Cox analysis was applied to analyze the prediction of the risk model and clinical parameters such as age, gender, T stage, N stage, M stage, and AJCC stage. To determine whether the risk model was affected by other clinical factors, multivariate Cox regression survival analysis was employed.

### GO and KEGG analyses for risk score-related genes

Genes showing a significant negative correlation with risk score were identified by cut-off criteria of Pearson | *R* |> 0.4 and *P* < 0.05 and then analyzed with GO [23] and KEGG [24] using the R package “clusterProfiler” [25].

### Comparison of immune-related characteristics between high- and low-risk score

ESTIMATE was used to calculate immune score and stromal score to determine the ratio of immune components to matrix components in TME. In addition, the infiltration score of immune cells in TME of the high-risk group and low-risk group was calculated by CIBERSORT [26].

### Prediction of immune/chemotherapy response

Immunotherapy and chemotherapy responses of LUAD cases were assessed by the Genomics of Drug Sensitivity in Cancer (GDSC) [27]. The Tumor Immune Dysfunction and Exclusion (TIDE) algorithm was employed to assess the response of each LUAD sample to immunotherapy. Unsupervised subclass mapping method SubMap [28] was used to evaluate the correspondence or similarity between risk groups in the TCGA dataset and patients receiving immunotherapy in the GSE78220 dataset. The diversity of chemosensitivity between high- and low-risk scores was analyzed by the Wilcoxon test.

### Statistical analysis

Statistical analyses were performed in R software (version 3.6.3). Chi-squared tests and Fischer’s exact tests were conducted for comparing inter-group discrete variables. Continuous variables within the two groups were compared using the Wilcoxon test. A comparison of more than two groups of continuous variables was performed using the Kruskal–Wallis test. Bilateral *P* < 0.05 was seen as statistically significant.

## Results

### Establishment of a prognostic gene signature with TGF-β signaling-related genes for LUAD patients

**Fig. 1:**
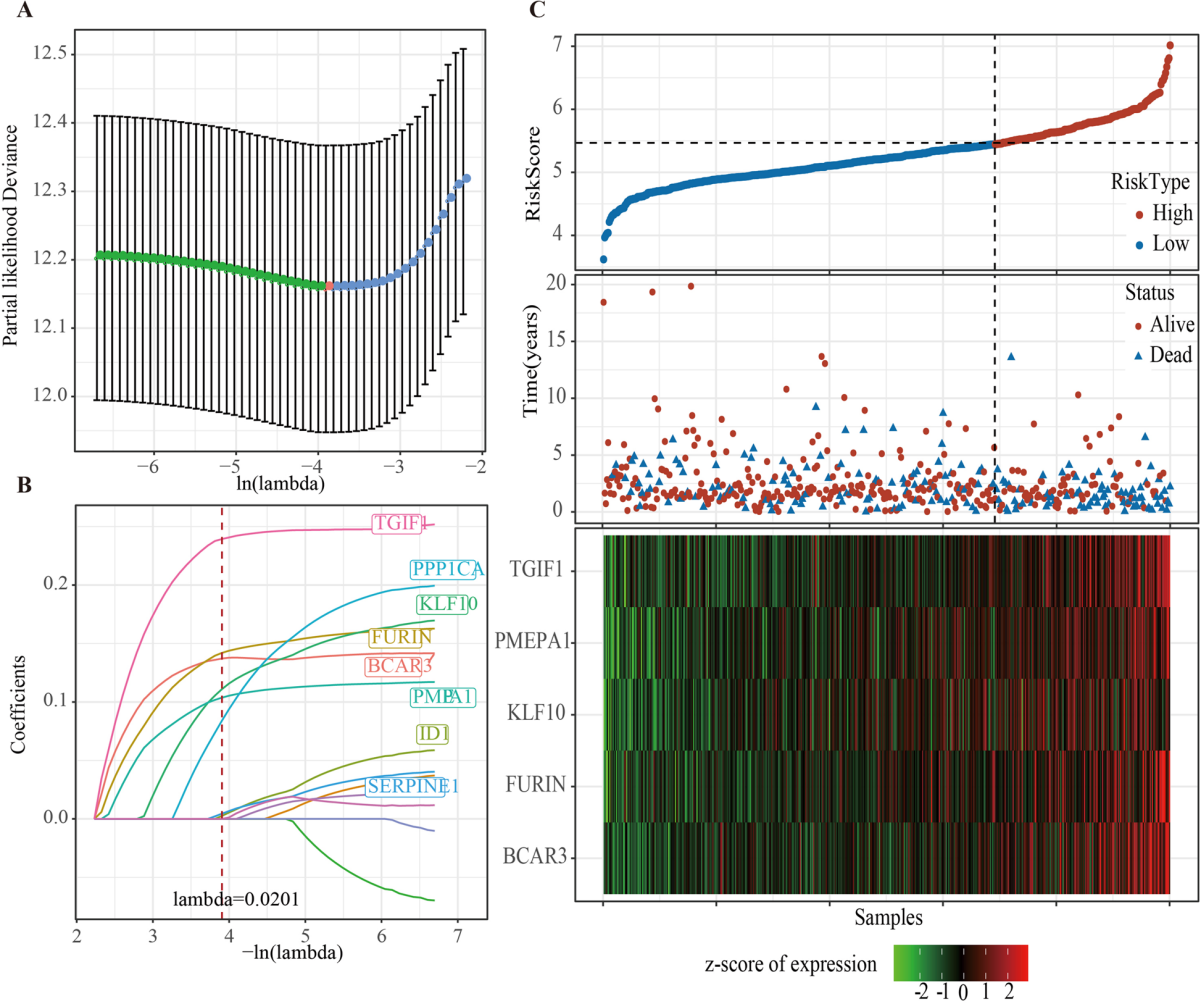
Establishment of a prognostic gene signature for LUAD patients based on TGF-β signaling-related genes.** A** LASSO Cox regression was applied to screen the optimal parameter with cross-validation. **B** The coefficients for each gene during the training process. **C** The risk score of each LUAD patient in TCGA was ranked in ascending order, survival status, and heat map of 5 TGF-β signaling-related gene expression profiles

The univariate Cox regression OS analysis showed that 13 TGF-β signaling-related genes were closely associated with the OS of LUAD patients (Supplementary Table S1). Eight genes were screened by LASSO and multivariate Cox regression and used to develop risk score signature (Fig. 1A, B). To reduce unnecessary component genes in the model, the stepAIC method was used to optimize the model. The risk score formula of LUAD patients was obtained: risk score = 0.126*PMEPA1 + 0.294*TGIF1 + 0.184*FURIN + 0.162*BCAR3 + 0.187*KLF10. The TCGA samples were arranged in ascending order according to the value of the risk score. We analyzed the survival times of samples in the high- and low-risk groups and found that the mean survival probability for patients with high-risk scores was lower than that for those with low-risk scores. The expression profiles of the five genes were shown in a heatmap, which revealed that the expression of the five genes was upregulated with the increase of risk score (Fig. 1C). In addition, we also compared the expression differences of these five genes in cancer and adjacent tumors. It can be observed that except BCAR3, PMEPA1, TGIF1, FURIN, and KLF10 were significantly overexpressed in tumor samples (Supplementary Fig. 2A). Survival analysis showed that the samples with a high expression of these genes had a poor prognosis (Supplementary Fig. 2B-F).

### Validation of the risk score signature

**Fig. 2:**
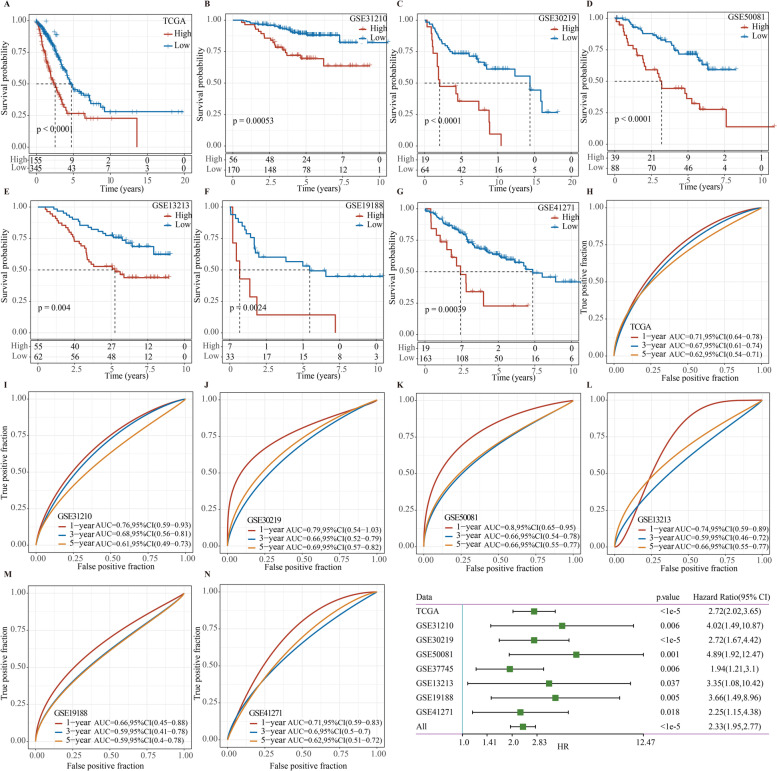
Verification of the effectiveness of the prediction model. Kaplan–Meier survival curve of different cohorts, including training set (**A**), GSE31210 (**B**), GSE30219 (**C**), GSE50081 (**D**), GSE13213 (**E**), GSE19188 (**F**), and GSE41271 (**G**) cohorts. Kaplan–Meier curve between high- and low-risk groups in TCGA-LUAD (**H**), GSE31210 (**I**), GSE30219 (**J**), GSE50081 (**K**), GSE13213 (**L**), GSE19188 (**M**), and GSE41271 (**N**) cohorts. **O** Univariate Cox analysis was employed to evaluate the correlation between risk score and prognosis of LUAD in each cohort

Survival analysis on the training set (TCGA-LUAD cohort) and external validation sets (GSE31210, GSE30219, GSE50081, GSE13213, GSE19188, and GSE41271 cohort) revealed that higher risk scores were closely linked to worse prognosis (Fig. 2A–G). The AUCs of the risk model for predicting 1-, 3-, and 5-year OS were 0.71, 0.67, and 0.62 in the training set, respectively (Fig. 2H). The AUCs of ROC curves in the validation sets were 0.76, 0.79, 0.8, 0.74, 0.66, and 0.71 for predicting 1-year OS, respectively. The AUCs of the ROC curves for 5-year OS in the validation sets were 0.61, 0.69, 0.66, 0.66, 0.59, and 0.62, respectively (Fig. 2I–N). Subsequently, univariate Cox analysis further confirmed the correlation between each cohort and LUAD prognosis (Fig. 2O). Overall, the results indicated the effectiveness of the prediction model.

### Association between the risk score and clinical characteristics

**Fig. 3:**
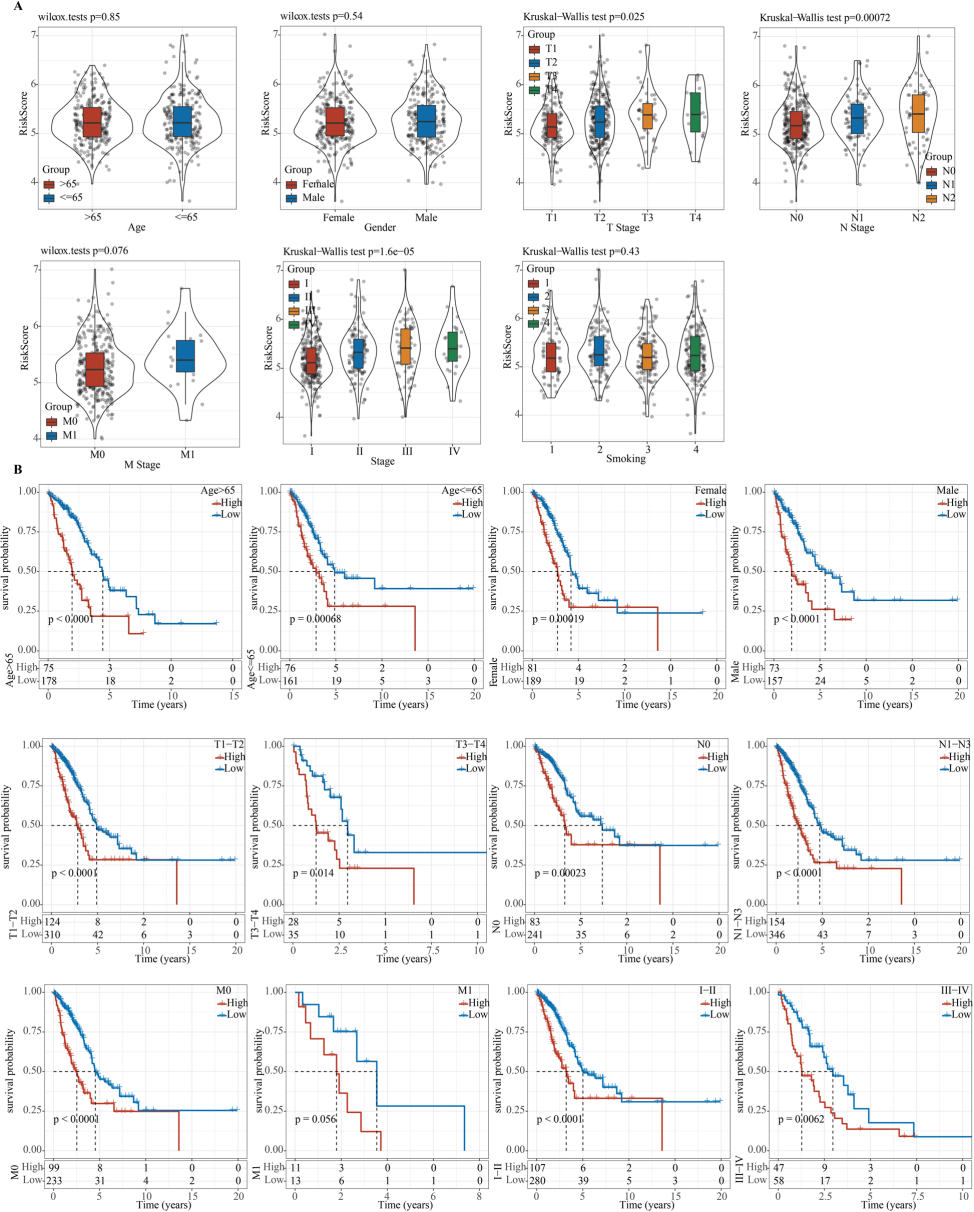
Correlation between the risk score and clinical characteristics. **A** Relationship between risk score and age, gender, T stage, N stage, M stage, AJCC stage, and smoking history. **B** OS Kaplan–Meier curves for LUAD samples stratified by age, gender, T stage, N stage, M stage, and AJCC stage

**Fig. 4:**
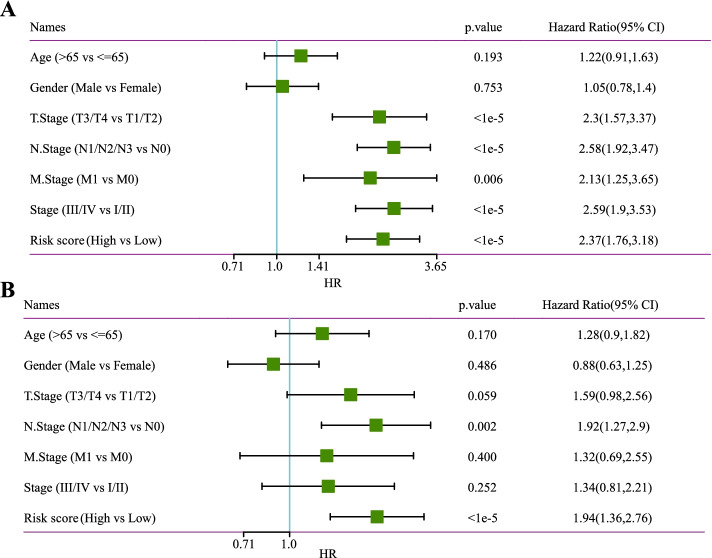
Recognition of independent prognostic factors. **A** Univariate Cox regression analysis on patients’ OS in the TCGA-LUAD cohort. **B** Multivariable Cox regression to analyze the correlation between risk score and clinical characteristics

We explored the risk scores in different subgroups stratified by age, gender, T stage, N stage, M stage, AJCC stage, and smoking history, respectively, and found that risk scores were not significantly linked to age, gender, M stage, or smoking, but were significantly related to T stage, N stage, and AJCC stage. Moreover, patients with advanced LUAD had noticeably higher risk scores than those with early LUAD (Fig. 3A). To better assess the prognostic ability of the risk model, we conducted a stratified OS analysis based on clinical risk factors. The model performed well in stratifying age > 65 and ≤ 65, male and female, T1–T2 and T3–4, N0 and N1–N3, M0, and AJCC stages I–II and III–IV (Fig. 3B). Furthermore, univariate and multivariate Cox regression analyses revealed that the N stage and the current model were independent predictors for LUAD prognosis (Fig. 4A, B). Taken together, the risk model established in this study had a high precision in predicting the OS of LUAD patients with different clinical characteristics.

### Identification and functional annotation of risk score-related genes

**Fig. 5:**
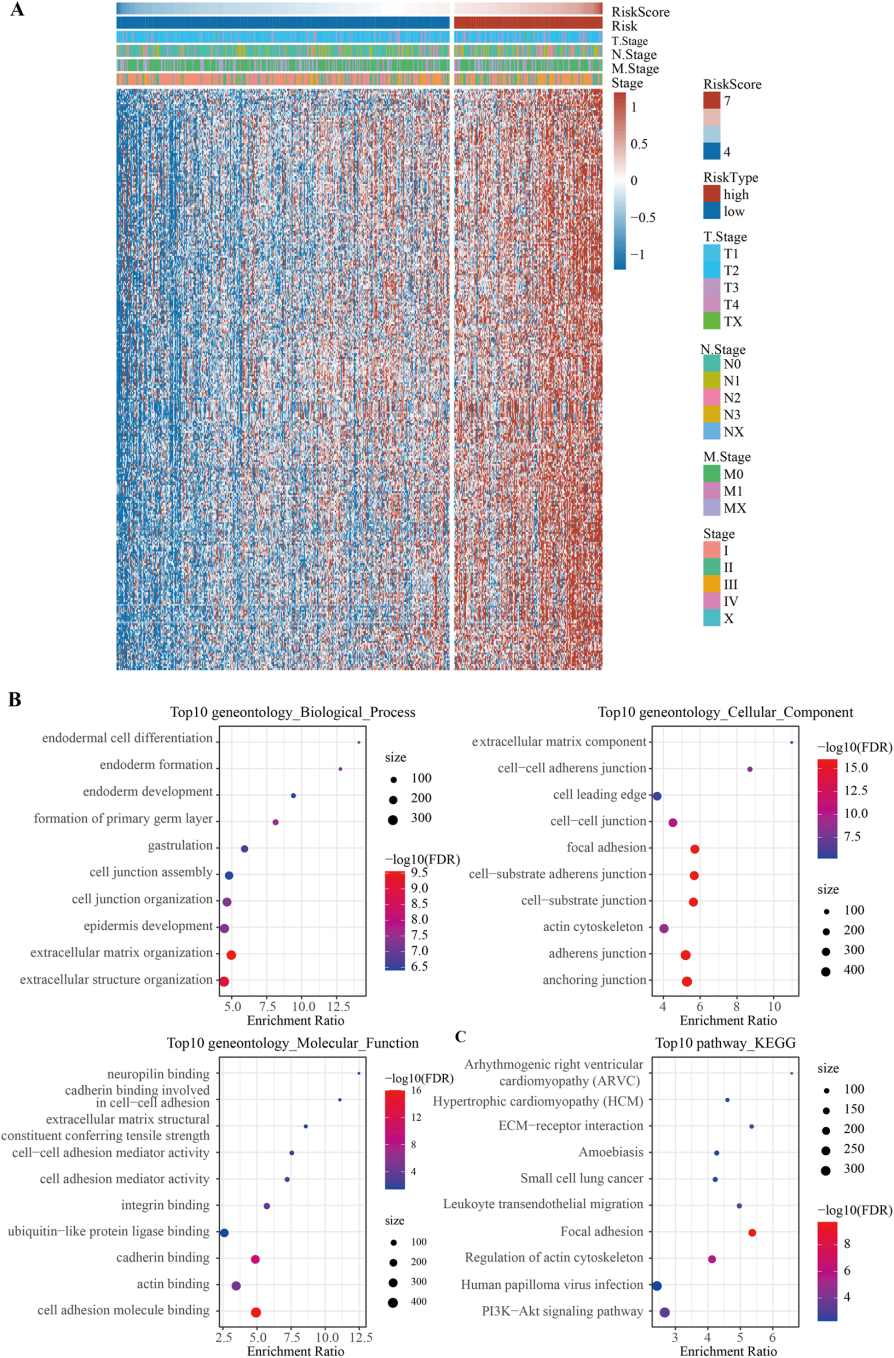
Identification and functional annotation of risk score-related genes. **A** Heat map showed the expression of risk score-related genes. **B** The bubble chart showed the result of the GO analysis of risk score-related genes. **C** KEGG enrichment analysis of ARGs. Top 10 significant KEGG signal pathways of risk score-related genes

A total of 299 genes with a significant negative correlation with risk score were identified by Pearson correlation analysis (Supplementary Table S2), and the heatmap of their expression is shown in Fig. 5A. To detect signal pathways of risk score-related genes, GO and KEGG enrichment analysis was carried out. In biological process (BP), enriched pathways such as endodermal cell differentiation, endoderm formation, and endoderm development were closely related to tissue development. The results showed that cellular component (CC) and molecular function (MF) of the risk score-related genes were involved in cancer cell migration (Fig. 5B). Moreover, KEGG enrichment analysis demonstrated that 299 genes were closely associated with ECM − receptor interaction, small cell lung cancer, and leukocyte transendothelial migration and so on (Fig. 5C).

### Immune cell infiltration and inflammation between high- and low-risk score

**Fig. 6:**
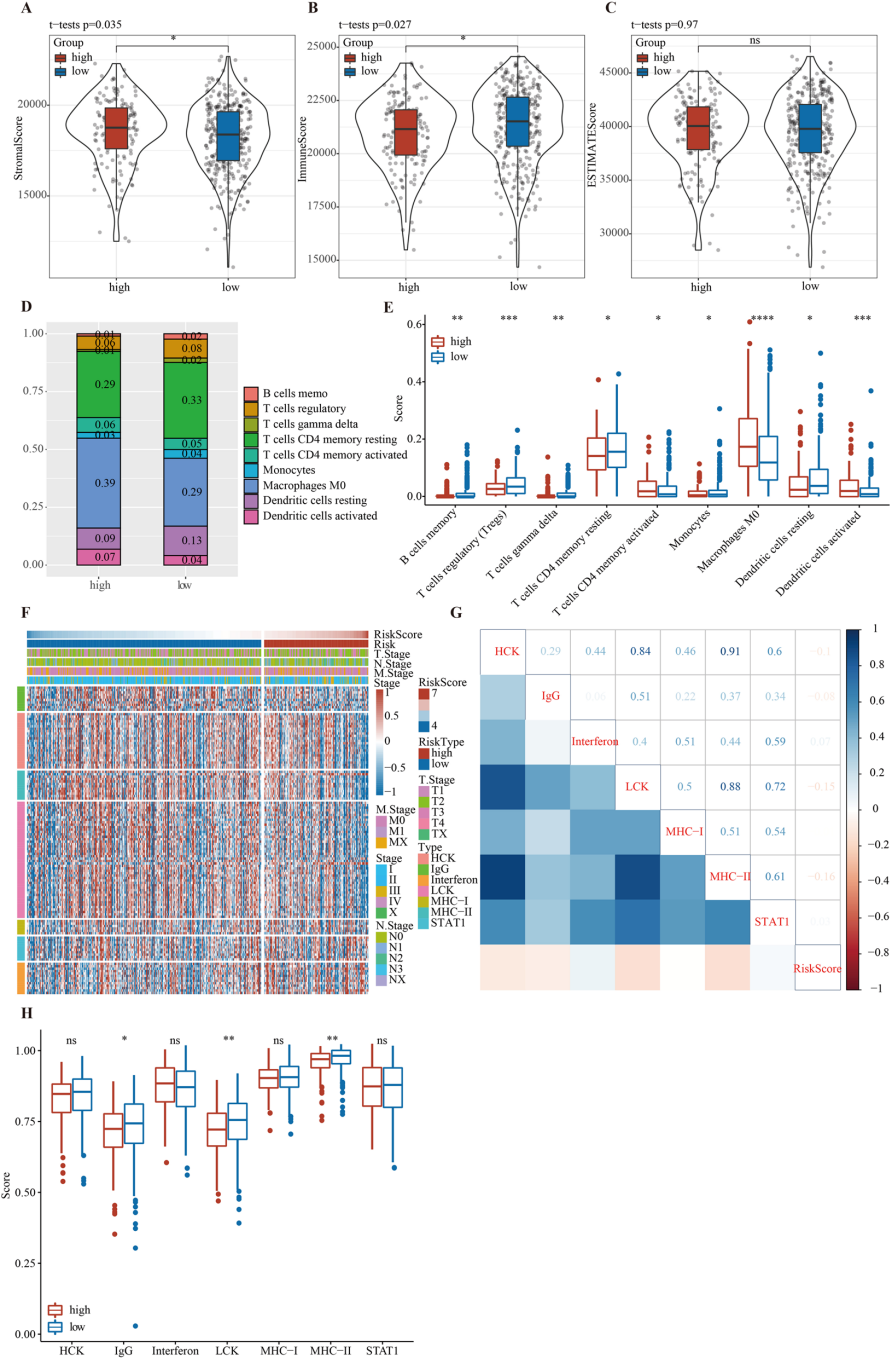
Immune cell infiltration and inflammation between high- and low-risk scores. **A**–**C** Stromal score, immune score, and ESTIMATE score between the high-risk score group and low-risk score group. **D** Boxplot showed the infiltration ratio of 9 immune cells in high- and low-risk groups. **E** Boxplot of infiltration scores of 9 kinds of immune cells in high- and low-risk groups. **F** Heat map displayed the relationship between risk score and 7 metagenes. **G** Correlation matrix of risk score and the seven metagenes. **H** Boxplot of the correlation between risk score and 7 metagenes

To determine the difference of TME status between the high-risk group and low-risk group, ESTIMATE analysis was carried out. The stromal score of the high-risk group was higher than that of the low-risk group (Fig. 6A). The low-risk score group showed a higher immune score (Fig. 6B), and there was no prominent diversity in ESTIMATE score between the two groups (Fig. 6C). Furthermore, in TME, 9 out of 22 immune cells, including memory B cells, regulatory T cells, gamma delta T cells, resting memory CD4 T cells, activated memory CD4 T cells, monocytes, macrophages M0, resting dendritic cells, and activated dendritic cells, showed significantly different infiltration ratios between high- and low-risk groups. Among these 9 kinds of cells, resting memory CD4 T cells and macrophages M0 accounted for the high proportion of TME in both high-risk and low-risk groups (Fig. 6D). The infiltration score of activated memory CD4 T cells, macrophages M0, and activated dendritic cells was significantly higher in the high-risk group, but that of memory B cells, regulatory T cells, gamma delta T cells, resting memory CD4 T cells, and monocytes and resting dendritic cells was significantly lower in the high-risk group than the low-risk group (Fig. 6E). To investigate the characteristics of tumor inflammation associated with risk score, we used 7 metagenes, including 104 genes linked to different types of inflammation and immune responses [29]. The heatmap presented the relationship between these genes and the risk score (Fig. 6F). The expression data of these metagenes were converted into enrichment scores by GSVA, and correlograms were generated based on comparisons between the risk score and the 7 metagenes. The results showed that risk score was negatively associated with MHC II, LCK, IgG, and HCK, which also scored higher in the low-risk score group (Fig. 6G, H).

### Prediction of response to immunotherapy, chemotherapy, and targeted therapy based on the risk model

**Fig. 7:**
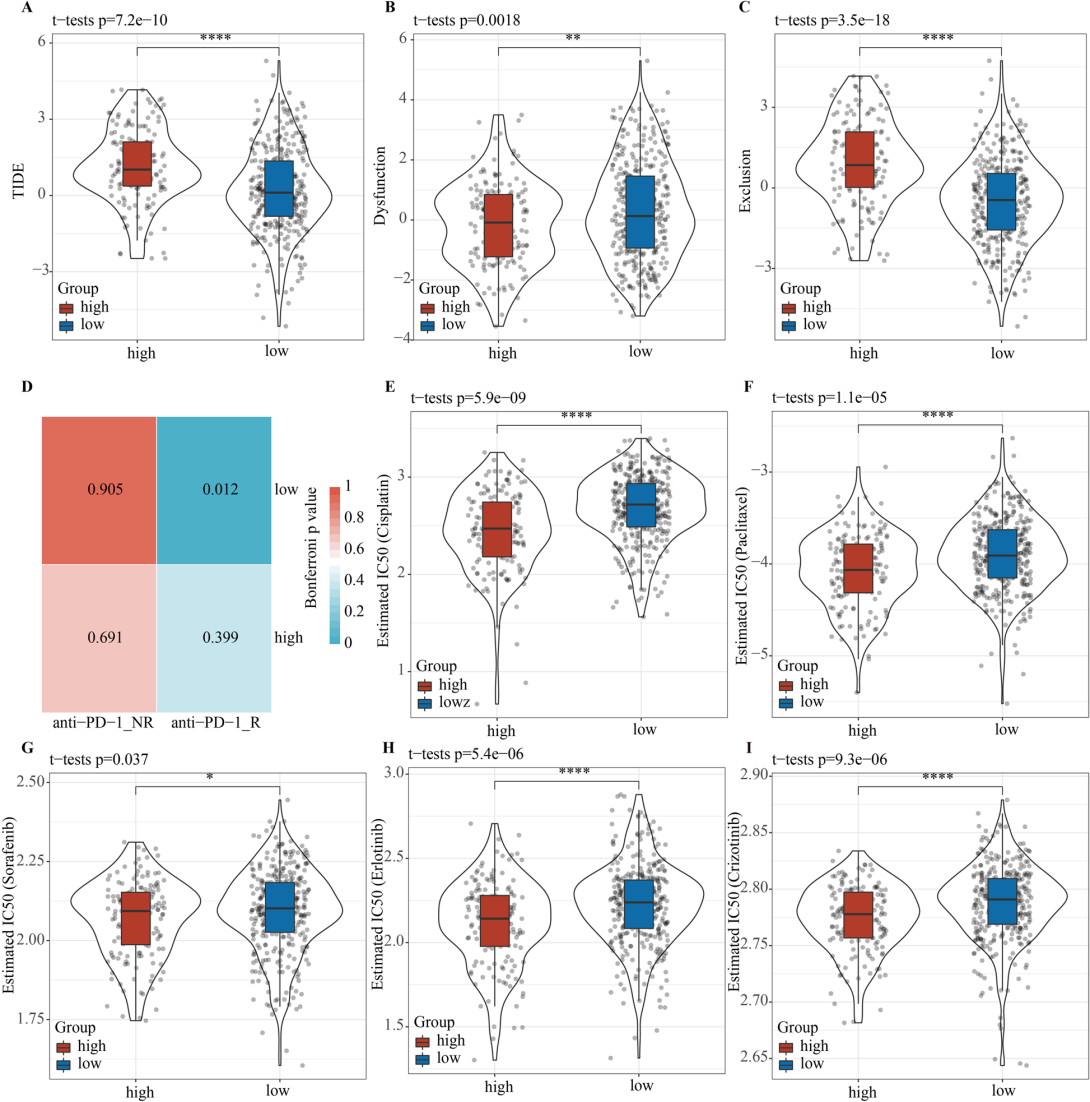
Predictive value of risk score for immunotherapy, chemotherapy, and targeted therapies. **A** Violin plots visualized the TIDE score between high- and low-risk groups. **B** Differences in T cell exclusion among patients with different risks. **C** Violin plots of the T cell dysfunction value for high- and low-risk groups. **D** Subclass mapping analyzed the response to anti-PD-1 treatment between high- and low-risk groups. Violin plots exhibited the diversity in estimated IC50 values of cisplatin (**E**), paclitaxel (**F**), erlotinib (**G**), sorafenib (**H**), and crizotinib (**I**) between high- and low-risk groups

We also explored the risk score in predicting the outcome of patients receiving immunotherapy, chemotherapy, and targeted therapy. Firstly, the TIDE algorithm was used to estimate the response of each risk group to immunotherapy. The low-risk group showed lower TIDE score and T cell exclusion score and higher T cell dysfunction score when compared with the high-risk group, suggesting that the immunotherapy response of low-risk patients may be more active (Fig. 7A–C). Submap analysis results indicated that the low-risk in melanoma patients from GSE78220 had a greater tendency to respond to anti-PD-1 treatment (Fig. 7D). At present, targeted therapy and chemotherapy are still the main treatment options for treating LUAD [30]; we therefore evaluated the sensitivity of the two risk groups to chemotherapeutic agents (cisplatin and paclitaxel) and targeted agents (erlotinib, sorafenib, and crizotinib). A comparison of the estimated IC50 values of each sample demonstrated that patients in the high-risk group were more sensitive to cisplatin, paclitaxel, erlotinib, sorafenib, and crizotinib (Fig. 7E–I). Hence, the risk score model can be used to predict the response of patients with LUAD to immunotherapy, chemotherapy, and targeted therapy.

## Discussion

The treatment of LUAD patients is mainly based on clinical indicators such as the TNM stage. However, increasing available treatment options also makes it difficult to decide on treatment plans [31]. In recent years, bioinformatics analysis using microarray technology has been proven to be an important tool in facilitating clinical decision-making [32]. Up to now, establishing and verifying predictive models allows studies to apply transcriptomic data and bioinformatics to improve the diagnosis, treatment, and prognosis of cancer [33–35]. It is reported that TGF-β signal transduction disorder is common in tumors and that inhibition of TGF-β signal is considered to be a prerequisite and a main way to improve the efficacy of immunotherapy, including in tumors with non-TGF-β-responsive cancer cells [36]. Accordingly, a comprehensive understanding of the expression profile of TGF-β signaling-related genes in LUAD may improve the diagnosis, treatment, and prognosis of patients.

In view of the biological effects of TGF-β signaling in cancer, we selected TGF-β signaling-related genes expressed in LUAD and developed a prognostic score model on the basis of 5 TGF-β signaling-related genes. LUAD patients with high-risk scores had shorter OS times than patients with low-risk scores. The same result was also found in the external data, reflecting the precision of the risk model in distinguishing LUAD with different prognoses. In addition, stratified analysis and multivariate Cox analysis confirmed that the risk score model also had a strong and independent predictive capacity when LUAD patients were re-grouped according to different clinicopathological characteristics.

It should be noted that most of the genes in the risk model have been identified to be associated with TGF-β signaling and are involved in regulating cancer progression. PMEPA1 is a direct target gene for TGF-β signaling and controls the duration and intensity of TGF-β/Smad signal transduction via a negative feedback loop [37]. It is reported that TMEPAI is high-expressed in many types of cancers except prostate cancer and is concerned with a poor prognosis [38]. PMEPA1 promotes EMT-mediated metastasis by activating TGF-β non-classical signal cascades in colorectal cancer [39]. The TMEPAI expression in lung cancer is positively correlated with mesenchymal phenotype and migration potential [40]. We found that TMEPAI was a risk gene in LUAD, which was consistent with the previous conclusion. It has been revealed that TGIF1 is abnormally high-expressed in LUAD tissues, and this is closely related to a high proliferative activity of tumor tissues and poor prognosis of patients with LUAD [41]. FURIN has been shown to be high-expressed in various cancer types, including in lung cancer; moreover, the mRNA and protein levels of FURIN are associated with the invasiveness of lung cancer cell lines [42]. Furthermore, FURIN expression is a potential marker of lung cancer and therapeutic target [43, 44]. As a protective factor for multiple myeloma, high-expressed BCAR3 indicates a favorable prognosis [45]. However, in primary breast tumors, a relatively low level of BCAR3 expression is associated with poor distant metastasis-free survival and recurrence-free survival [46]. Similarly, our analysis showed that BCAR3 was a risk factor for LUAD. Previous studies of Vivek Kumar Mishra found that in non-small cell lung cancer, KLF10 suppresses TGF-β-induced EMT via a negative feedback mechanism [47]. The above evidence suggested that all the five TGF-β signaling-related genes were associated with malignant processes of many kinds of cancers, including LUAD.

According to a previous report, TGF-β signaling regulates inflammatory/immune cell infiltration in TME [48]. We found differences in TME status among LUAD patients with different risks not only in immune and matrix scores, but also in immune cell infiltration, which could further affect patients’ response to immune checkpoint blocking therapy. Recent studies have indicated that TME regulates tumor response to immunotherapy [49]. We therefore predicted the response of LUAD patients with different risks to immunotherapy and observed that low-risk patients had a higher tendency to respond to anti-PD-1 treatment and were more sensitive to chemotherapy and targeted therapy.

## Conclusions

This study developed a 5-gene signature on the basis of TGF-β signaling-related genes for predicting the prognosis of LUAD. It was proven that the risk scoring model had a strong and independent prediction ability. The current risk model can characterize the TME and can be used to predict the response of LUAD patients to immunotherapy, chemotherapy, and targeted therapy. A larger sample size is needed to further study the risk prediction model to validate its use in the clinical management of LUAD.

## Supplementary Information

**Additional file 1:** **Supplementary Figure 1.** Work flow chart.

**Additional file 2:** **Supplementary Figure 2.** Expression and prognosis of five genes. A: Differential expression distribution of five genes in cancer and adjacent samples. B-F: Prognostic K-M curve of high expression samples and low expression samples of 5 genes, The best cut-off value was obtained by maxstat to group the patients.

**Additional file 3:** **Supplementary Table S1.** Univariate Cox regression analysis of 54 TGFβ signaling-related genes.

**Additional file 4:** **Supplementary Table S2.** The genes identified by Pearson correlation analysis were significantly negatively correlated with risk score.

## Data Availability

The datasets generated during and/or analyzed during the current study are available in the [GSE31210] repository, [https://www.ncbi.nlm.nih.gov/geo/query/acc.cgi?acc=GSE31210]; in the [GSE30219] repository, [https://www.ncbi.nlm.nih.gov/geo/query/acc.cgi?acc=GSE30219]; in the [GSE50081] repository, [https://www.ncbi.nlm.nih.gov/geo/query/acc.cgi?acc=GSE50081]; in the [GSE13213] repository, [https://www.ncbi.nlm.nih.gov/geo/query/acc.cgi?acc=GSE13213]; in the [GSE19188] repository, [https://www.ncbi.nlm.nih.gov/geo/query/acc.cgi?acc=GSE19188]; in the [GSE41271] repository, [https://www.ncbi.nlm.nih.gov/geo/query/acc.cgi?acc=GSE41271]; in the [GSE31210] repository, [https://www.ncbi.nlm.nih.gov/geo/query/acc.cgi?acc=GSE31210]; in the [GSE30219] repository, [https://www.ncbi.nlm.nih.gov/geo/query/acc.cgi?acc=GSE30219]; in the [GSE50081] repository, [https://www.ncbi.nlm.nih.gov/geo/query/acc.cgi?acc=GSE50081]; in the [GSE13213] repository, [https://www.ncbi.nlm.nih.gov/geo/query/acc.cgi?acc=GSE13213]; in the [GSE19188] repository, [https://www.ncbi.nlm.nih.gov/geo/query/acc.cgi?acc=GSE19188]; and in the [GSE41271] repository, [https://www.ncbi.nlm.nih.gov/geo/query/acc.cgi?acc=GSE41271].

## Abbreviations

TME: Tumor microenvironment
LUAD: Lung adenocarcinoma
MSigDB: Molecular Signature Database
LASSO: Least absolute shrinkage and selection operator
OS: Overall survival
GEO: Gene Expression Omnibus
ROC: Receiver operating characteristic curve
GDSC: Genomics of Drug Sensitivity in Cancer
