# Supplementary figures and images for: Identification of a TGF-β signaling-related gene signature for prediction of immunotherapy and targeted therapy for lung adenocarcinoma
Source: World J Surg Oncol. 2022 Jun 6;20:183. doi: 10.1186/s12957-022-02595-1 (PMC9172180; doi:10.1186/s12957-022-02595-1)

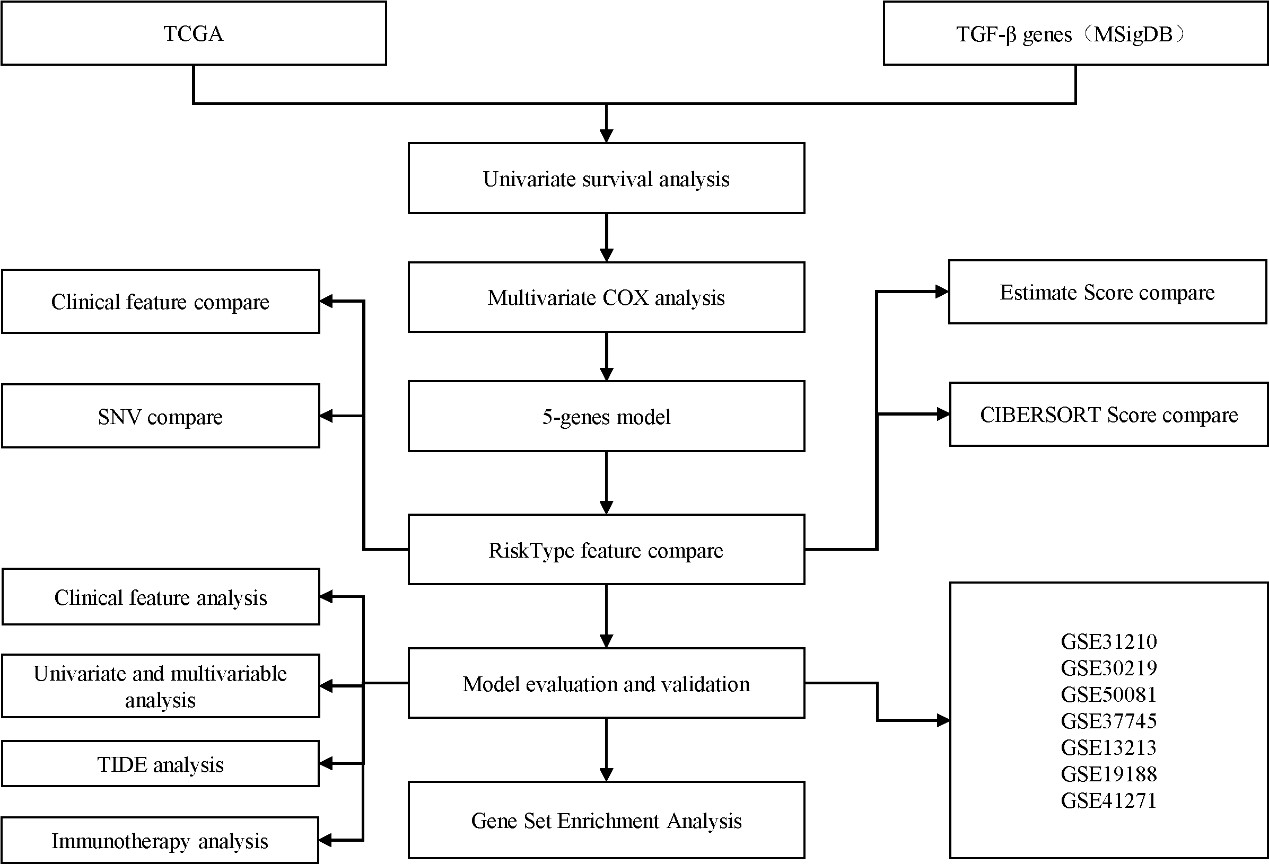

Supplement: Supplementary file 1 — Additional file 1: Supplementary Figure 1. Work flow chart. [file 12957_2022_2595_MOESM1_ESM.jpg]

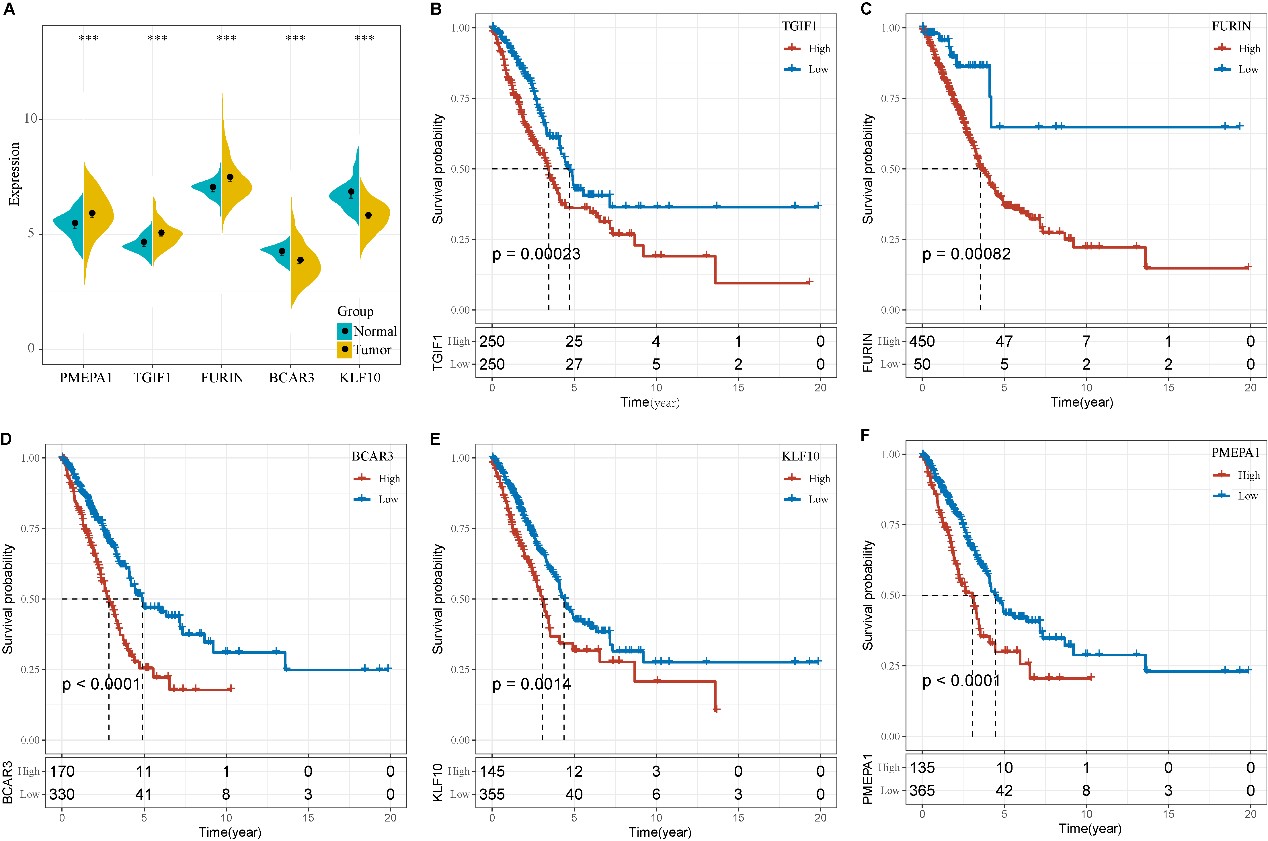

Supplement: Supplementary file 2 — Additional file 2: Supplementary Figure 2. Expression and prognosis of five genes. A: Differential expression distribution of five genes in cancer and adjacent samples. B-F: Prognostic K-M curve of high expression samples and low expression samples of 5 genes, The best cut-off value was obtained by maxstat to group the patients. [file 12957_2022_2595_MOESM2_ESM.jpg]
